# A consensus blood transcriptomic framework for sepsis

**DOI:** 10.1038/s41591-025-03964-5

**Published:** 2025-09-30

**Authors:** Brendon P. Scicluna, Kiki Cano-Gamez, Katie L. Burnham, Emma E. Davenport, Andrew Reese Moore, Soumen Khan, Charles J. Hinds, Olaf L. Cremer, Purvesh Khatri, Timothy E. Sweeney, Julian C. Knight, Tom van der Poll

**Affiliations:** 1 Department of Applied Biomedical Science, Faculty of Health Sciences, Mater Dei hospital, University of Malta, Msida, Malta; 2 Centre for Molecular Medicine & Biobanking, University of Malta, Msida, Malta; 3 Centre of Infection & Molecular Medicine, Amsterdam UMC, University of Amsterdam, Amsterdam, the Netherlands; 4 Centre for Human Genetics, Nuffield Department of Medicine, University of Oxford, Oxford, UK; 5 Wellcome Sanger Institute, Wellcome Genome Campus, Hinxton, UK; 6 Division of Pulmonary, Allergy and Critical Care Medicine, Stanford University, Stanford, CA USA; 7 Institute for Immunity, Transplantation and Infection, Stanford University, Stanford, CA USA; 8 Center for Biomedical Informatics Research, Department of Medicine, Stanford University, Stanford, CA USA; 9 William Harvey Research Institute, Barts and The London School of Medicine and Dentistry, Queen Mary University, London, UK; 10 Department of Intensive Care Medicine, University Medical Centre Utrecht, Utrecht University, Utrecht, the Netherlands; 11 Inflammatix Inc., Burlingame, CA USA; 12 Division of Infectious Diseases, Amsterdam UMC, University of Amsterdam, Amsterdam, the Netherlands

**Keywords:** Sepsis, Translational immunology, Diagnostic markers, RNA sequencing

## Abstract

Sepsis is a life-threatening condition driven by a maladaptive host response to infection. To establish a standardized blood transcriptomic subtype model, we aggregated blood transcriptomics data from two major sepsis cohorts: the Molecular Diagnosis and Risk Stratification of Sepsis (MARS) project (*n* = 678 sampled on intensive care unit admission; ClinicalTrials.gov registration no. NCT01905033) and the Genomic Advances in Sepsis (GAinS) study (*n* = 444 sampled on intensive care unit admission and *n* = 817 follow-up samples; ClinicalTrials.gov registration no. NCT00131196). We demonstrate a strong interconnection across three separate classification methods, resulting in the proposed groupings of three consensus transcriptomic subtypes (CTSs). The distinguishing characteristics of CTS1 included gene activation of typical inflammatory pathways, more pronounced endothelial activation and an overall immature neutrophil theme. CTS2 was characterized by gene activation of a heme metabolism pathway, fibrinolytic disturbances and platelet and eosinophil signatures. CTS3 was associated with genes involved in the activation of allograft rejection, interferon signaling and anticoagulation functions, together with lymphocyte and nonclassical monocyte features. Evaluating CTS classification in independent patient cohorts, specifically the vasopressin vs noradrenaline as initial therapy in septic shock (VANISH) randomized controlled trial (*n* = 176; ISRCTN registration no. ISRCTN20769191) and patients hospitalized with suspected sepsis at a district hospital in Uganda (*n* = 128), ascertained the robustness of our approach. Notably, post hoc analysis of a pseudo-randomized cohort, along with a reanalysis of the VANISH trial data, unmasked a harmful signal in CTS2-assigned patients treated with corticosteroids. The CTS classification method aligns diverse sepsis transcriptomic subgroupings into a robust, reproducible framework, thereby enabling biological interpretation and potentially assisting aspects of clinical trial design to advance precision medicine in sepsis.

## Abstract (web summary)

In a combined analysis of two large patient cohorts, three peripheral consensus transcriptomic subtypes of sepsis are identified, which can be linked to an 18-gene classifier associated with different odds of mortality and may offer a way to tailor care for patients with sepsis.

## Main

Sepsis is a potentially fatal clinical syndrome characterized by a maladaptive host response to infection, frequently associated with substantial long-term health issues for those who survive^1–3^. Despite our improved understanding of sepsis pathophysiology^4–6^, the heterogeneity of patient responses to infection remains a major challenge in developing specific treatment strategies. Several features of the host response that may be maladaptive in terms of degree of activation or dysregulation, have been described in sepsis, including inflammation, activation of the complement and coagulation systems, and enhanced apoptosis of particularly T lymphocytes^7^. Although these different reactions of the immune system provide us with an insight into sepsis immunopathology, they do not fully capture the complexity and heterogeneity of the host response in sepsis. To better understand this complexity, blood transcriptomics has enabled the identification of biologically distinct molecular subtypes^8–12^. A prevalent finding from the initial studies was the identification of a poor prognosis subtype, characterized by blood transcriptomes indicative of diminished innate and adaptive immune responses^8,9,11^. This subtype has been referred to by various nomenclatures arising from different patient classification methods, underscoring the existing challenges in the field. Notably, blood transcriptomic subtypes may be useful to predict the response to therapy^13^, which with increasing availability of immunomodulatory agents and evidence of benefit in severe coronavirus diseases 2019 (COVID-19)^14–16^ is crucial to identify patients who would benefit or be harmed when managing sepsis. Despite these inroads, the clinical implications of those findings are currently limited. The lack of a standardized subtype model is an important factor.

Disease stratification is commonly acknowledged to be substantially enhanced retrospectively by transcriptomics to discover and assign subtypes^17^. Blood transcriptomic profiling offers a comprehensive analysis of the circulating cellular immune and inflammatory host response in sepsis, revealing the complex interplay of multiple molecular pathways involving mainly white blood cells^18^. Although commonly studied, the effectiveness of blood transcriptomics in translating to clinical applications is hindered by inconsistent findings, particularly the varying number of subtypes identified by different research groups, currently ranging from two to four^8,9,11^. These discrepancies are probably due to variations in patient populations and management, sample preparation procedures, gene expression platforms, data processing and algorithms. In addition, overfitting to the data could also be a factor, at least to some extent. To address the lack of a definitive and widely accepted approach to conduct these studies, it is necessary to develop a comprehensive framework that incorporates and evaluates many methodologies. We envisioned that a comprehensive comparison of blood transcriptome subtype membership using different classification strategies applied to a common cohort could help resolve discrepancies in subtype numbers and their biological interpretation. Reaching a consensus on blood transcriptome subtypes is key to applying techniques that can assist in risk stratification, identifying diagnostic and prognostic biomarkers, and enhancing the design of clinical trials by classifying patients according to distinct pathophysiological pathways, that is, predictive enrichment^12^. Considering that blood transcriptomics is a high-throughput molecular assay closely linked to both cellular phenotypes and clinical presentation, we also wanted to define the key biological features that characterize subtypes, informed by other data sources, including single-cell RNA profiles and plasma proteomics, and determine if subtype assignment correlated with patient outcome and clinical severity. In doing so, we sought to create a paradigm for collaborative, community-based efforts that would make molecular subtypes easier to translate into the clinic for sepsis and possibly related critical illness syndromes.

## Results

### Identification of three consensus transcriptomic subtypes of sepsis with clinical implications

**Fig. 1:**
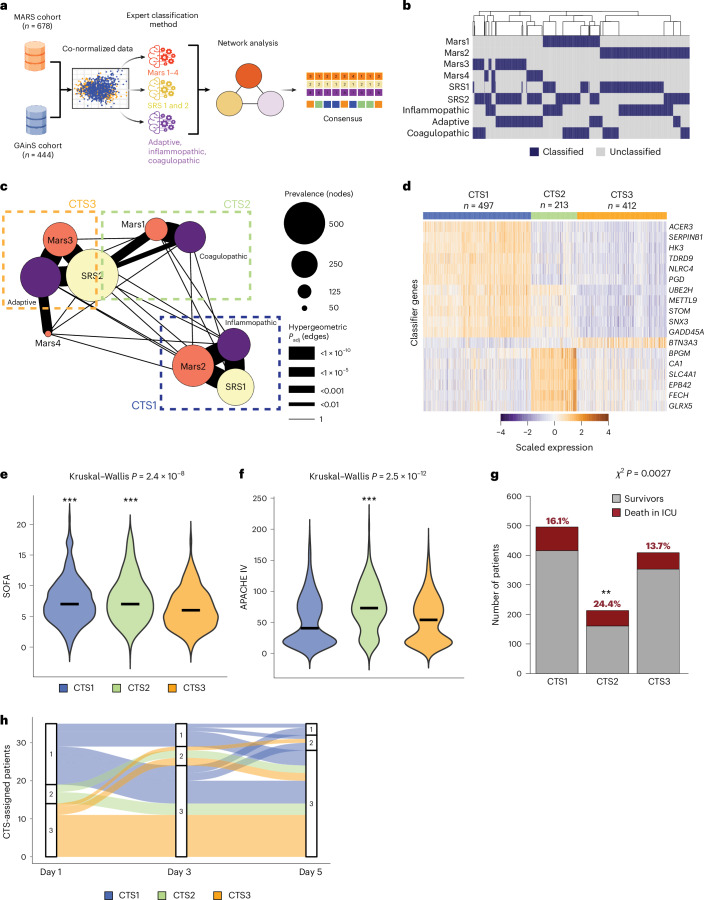
Identification of the CTSs of sepsis. **a**, Analytical workflow for the classification of large sepsis cohorts with blood transcriptomic data from ICU admission of the MARS cohort from the Netherlands and the GAinS cohort from the UK. After co-normalization, each expert group classified the common (co-normalized) dataset (*n* = 1,122 unique patients) using their clustering classifier genes. This was followed by concordance analysis of the three clustering platforms and application of a network analytical method to identify CTSs. **b**, Unsupervised clustering of patient samples classified as three different sepsis patient classification strategies illustrating the overlap between them in a binary heatmap representation. **c**, Network of sepsis subgroups across three classification systems. Each node corresponds to a single subtype colored according to expert group. The edges correspond to the Jaccard similarity coefficient. Edge width is inversely proportional to the adjusted *P* value from a one-sided hypergeometric test, with wider edges indicating greater statistical significance. The colored spheres indicate subtype assignment according to previously published algorithms (orange, MARS; yellow, GAinS (SRS); purple, Stanford (inflammopathic, coagulopathic and adaptive)). The three primary clusters, identified from Markov clustering, are highlighted and annotated as CTS1–3. **d**, Heatmap representation of the 18 most significant genes that discriminate CTSs using a Kruskal–Wallis test (Benjamini–Hochberg-adjusted *P* < 0.01) and random forest classifier. **e**,**f**, Violin plot illustrating SOFA (**e**) and APACHE IV (**f**) scores stratified into CTSs 1–3. **g**, Bar plot of ICU mortality stratified according to CTS membership analyzed using a chi-squared post hoc test. **h**, Alluvial plot illustrating the trajectories of 35 patients with sepsis sampled at ICU admission (day 1) and after 3 and 5 days of ICU stay, categorized into CTS1–3. Dunn’s or chi-squared post hoc test *P* values of **P < 0.01 and ***P < 0.001 relative to CTS3; for APACHE IV, scores relative to CTS1 are also shown.

We combined the blood transcriptomic data of two large prospective observational studies in critical illness caused by sepsis from the Molecular Diagnosis and Risk Stratification of Sepsis (MARS) project in the Netherlands (clinicaltrials.gov registration no. NCT01905033) and the UK Genomic Advances in Sepsis (GAinS) study (clinicaltrials.gov registration no. NCT00131196). A total of 1,122 unique patient samples obtained on the day of intensive care unit (ICU) admission (MARS *n* = 678; GAinS *n* = 444) and blood transcriptomes generated using microarray (*n* = 834) or RNA sequencing (RNA-seq) (*n* = 288) were included in this study^8,9,19–21^. In addition, the GAinS researchers collected 817 samples obtained 3 or 5 days after ICU admission. To minimize technical variability, normalized RNA-seq and microarray datasets were adjusted for batch, and microarray probe sequences were re-annotated against the current genome build (GRCh38). Using this combined dataset, each of the three investigator groups who previously published on the blood transcriptomic subtypes of sepsis^8–11^, independently used their subtype classification algorithm to classify samples from ICU admission. Therefore, each patient sample had three subtype classifications: sepsis response signatures ‘SRS1 and SRS2’^8^; ‘Mars1–4’^9^; ‘inflammopathic’, ‘adaptive’ and ‘coagulopathic’^11^. The analytical workflow is depicted in Fig. 1a. We used a network-based method to examine the correlation between the three sepsis subtype classification systems, each comprising 2–4 subtypes (Fig. 1b). The distance network was then clustered using a Markov clustering algorithm^22^. Network granularity was controlled by a standard inflation factor; silhouette widths were used as a measure of clustering robustness and classifier performance^23^. The overlap between sepsis subtype classifications was determined using hypergeometric tests on the union sets, adjusting the nominal probabilities with the Benjamini–Hochberg’s method^24^. On the basis of the most effective inflation factor, we identified three clearly defined consensus transcriptomic subtypes (CTSs) that exhibited substantial interconnectedness across the three distinct subtype classification methods (Fig. 1c and Table 1). The SRS1, Mars2 and inflammopathic subtypes clustered together (CTS1); SRS2 clustered with the Mars1 and coagulopathic subtypes (CTS2), as well as the Mars3 and adaptive subtypes (CTS3). Patients classified as Mars4 did not cluster together in this analysis; however, a hypergeometric test yielded a substantial association with ‘adaptive’ classification (Fig. 1c). The low prevalence of Mars4 patients may explain the lack of a robust cluster.

**Table 1:** Clinical characteristics of patients with sepsis classified as CTSs 1–3 from the MARS and GAinS cohorts sampled on the day of ICU admission

	CTS1	CTS2	CTS3	*P*
Patients, *n*	497	213	412	
Demographics				
Age, mean (s.d.)	61.4 (15.6)	62.4 (13.6)	60.3 (15.9)	0.238^a^
Sex, males (%)	265 (53.3)	118 (55.4)	258 (62.6)	0.01^b^
Chronic comorbidity, *n* (%)				
COPD	62 (12.5)	29 (13.6)	64 (15.5)	0.726^b^
Diabetes	62 (12.5)	44 (20.7)	61 (14.8)	0.806^b^
Malignancy	31 (6.2)	13 (6.1)	19 (4.6)	0.091^b^
Site of infection, *n* (%)				
Lung	222 (44.8)	98 (46.0)	285 (69.7)	<0.001^b^
Abdominal	220 (44.4)	65 (30.5)	43 (10.5)	<0.001^b^
Other^c^	55 (11.1)	50 (23.5)	84 (20.4)	<0.001^b^
Severity of disease on ICU admission				
APACHE IV score, median (Q1–Q3)	40.5 (18–81)	73 (50–100)	54 (19–79)	<0.001^d^
SOFA score, median (Q1–Q3)	7 (5–9)	7 (5–10)	6 (4–8)	<0.001^d^
Septic shock, *n* (%)	175 (35.2)	73 (34.3)	50 (19.4)	<0.001^b^
Outcome				
Length of ICU stay, median days (Q1–Q3)	4 (2–8)	6 (2–12)	5 (3–9.75)	0.038^d^
28-day mortality, *n* (%)	80 (16.1)	61 (28.6)	65 (15.8)	0.003^b^

^a^Two-sided analysis of variance (ANOVA). ^b^Chi-squared test after Bonferroni correction. ^c^Other sites of infection, genitourinary, skin, primary bacteremia, mediastinitis. ^d^Kruksal–Wallis test. COPD, chronic obstructive pulmonary disease.

**Extended Data Fig. 1:**
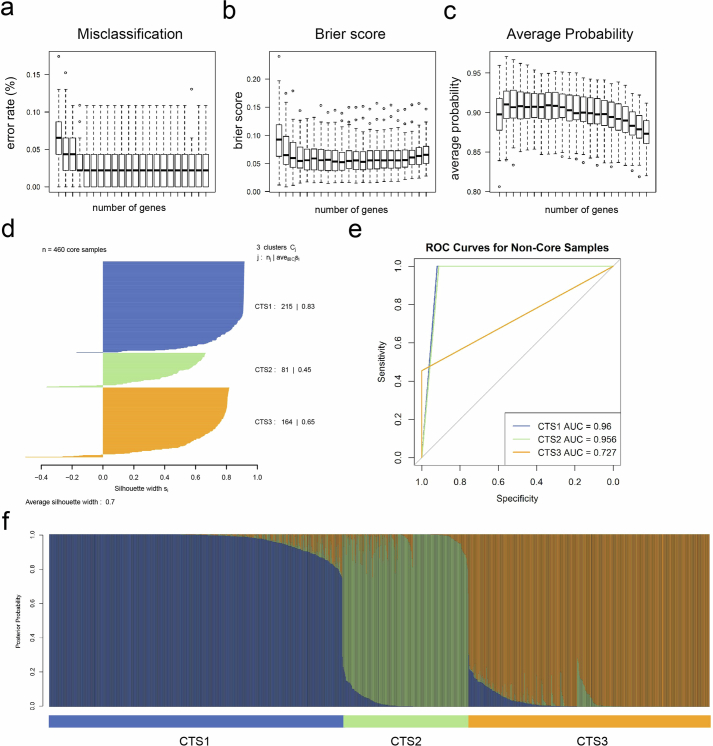
Derivation of a consensus transcriptomic subtype (CTS) gene expression classifier. (**a**) Misclassification error rate as a function of incremental addition of genes to the random forest classifier. (**b**) Brier scores that measure the mean squared difference between predicted probabilities assigned by the random forest classifier and the actual CTS assignment. Lower Brier scores indicates better predictions. (**c**) Average predicted probability of the specific subtypes across all the individual trees in random forest considering incremental addition of genes to the classifier. Higher probabilities indicate greater confidence in classifying CTSs. Boxplots represent the distribution of (**a**) misclassification error rates, (**b**) Brier scores, and (**c**) average probabilities for classifiers built with an incrementally increasing number of genes. Performance metrics were derived using a 10-fold cross-validation procedure within the combined training cohort. The unit of study was the individual patient. The analysis was performed on a total of 460 independent biological samples (core patient samples) obtained on ICU admission from the MARS and GAinS cohorts. Each boxplot summarizes the performance across the 10-fold cross-validation, where the central line indicates the median, the box represents the interquartile range (IQR), and the whiskers extend to 1.5 times the IQR. (**d**) Silhouette width analysis of the 460 core samples assigned to CTS1, CTS2, or CTS3, illustrating overall model fit and cluster stability. (**e**) Receiver-operator-characteristic (ROC) area-under-the-curve (AUC) depicting the performance of “non-core” samples in classifying CTSs. CTS3 classification was not favorable relative to CTS1 or 2, exemplified by the low AUC (0.727). (**f**) Posterior probabilities of all patient samples assigned to CTS1, CTS2, or CTS3 calculated from a random forest machine learning classifier.

Our network-based analysis identified a group of core consensus samples, which are representative of each CTS. Core samples were defined by correspondence between the subtype assigned initially by the research group and the subtype associated with each CTS. These samples showed a strong agreement in their subtype membership across distinct subtyping methods, as determined using a hypergeometric test (*P* < 0.05). We derived a classification system for predicting CTSs using gene expression data from core samples. Genes were ranked according to their importance in discriminating CTSs in the core sample set using a Kruskal–Wallis test and tenfold cross-validation in a one-versus-all scheme. Subsequently, a random forest classifier consisting of 500 trees was used to assess the performance of the classifier in terms of misclassification error rates, Brier scores and average probabilities (Extended Data Fig. 1a–c). We settled on an 18-gene panel (Fig. 1d), with an out-of-bag error rate equal to 2.2%. Silhouette width analysis yielded a robust model fit with an average width equating to 0.7 (Extended Data Fig. 1d). The receiver operating characteristic (ROC) area under the curve (AUC) for non-core samples further supports our approach of identifying core samples, particularly because of the lower discriminative power of CTS3 for non-core samples (Extended Data Fig. 1e). Moreover, quantification of the probability distribution output using the random forest prediction model for each sample (*n* = 1,122) illustrated confident assignments to each subtype with overall probabilities of more than 0.8 (Extended Data Fig. 1f).

**Extended Data Fig. 2:**
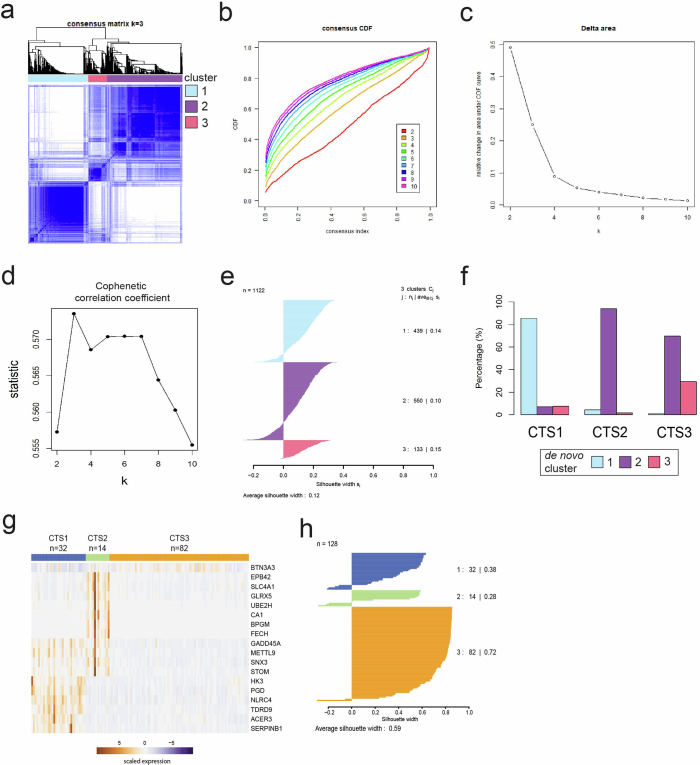
De novo consensus clustering and concordance analysis. (**a**) Consensus clustering heatmap depicting the result of de novo clustering of all patient samples from ICU admission (n = 1122) using the entire transcriptome (n = 7260 unique genes) specifically at the optimal k (subtype number) of 3 (**b**) Consensus cumulative distribution function (CDF) plot at each evaluated k (2-10), (**c**) the area-under-CDF-curve illustrating that beyond k = 3 there is no appreciable change in AUCDF, and (**d**) cophenetic correlation coefficients across k = 2 to k = 10, with a clear peak at k = 3 further reinforcing a three subtype model. (**e**) Silhouette width analysis and plot showing overall positive widths per CTS and on average, indicating optimal clustering. (**f**) Barplots depicting the percentage of patients assigned to CTSs that cluster to de novo clusters 1-3. (**g**) Heatmap plot of CTS gene classifier and CTS assigned patients of the Research in the Epidemiology of Severe and Emerging Infections in Uganda (RESERVE-U) study (BioProject: PRJNA794277). (**h**) Silhouette width analysis depicting positive widths throughout subtypes and on average, exemplifying the optimal performance of the CTS gene classifier in assigning RESERVE-U patients to CTS1-3.

**Extended Data Fig. 3:**
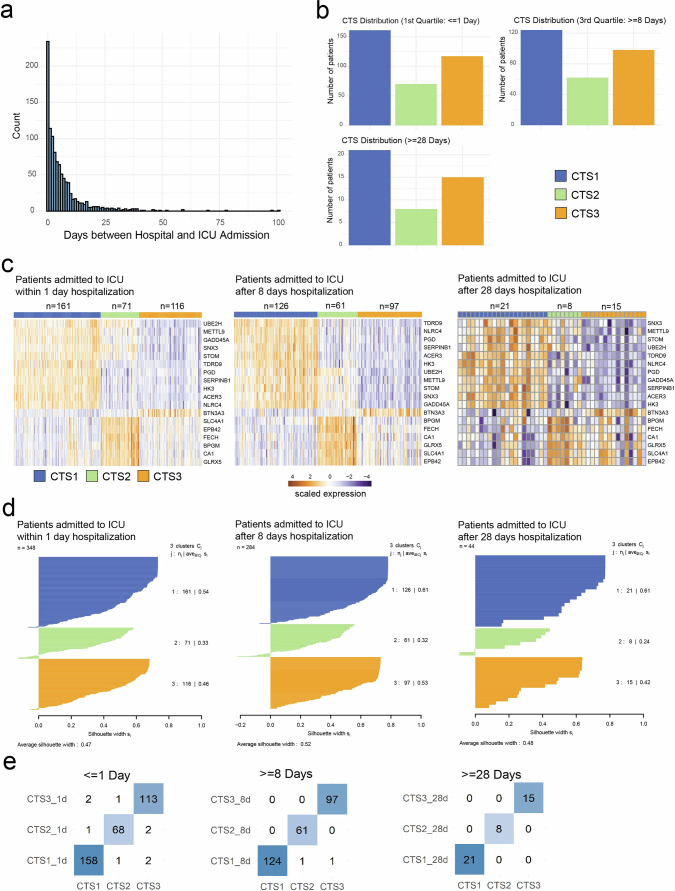
Evaluation of the association between consensus transcriptomic subtype (CTS), clinical severity, and outcome in patient cohorts. (**a**) Histogram plot illustrating the distribution of the interval between hospital admission and ICU admission in days. (**b**) Barplots depicting the distribution of CTS assigned patients considering the first quartile (Q1) of hospital to ICU admission interval ( ≤ 1 day), third quartile (Q3) equating to ≥ 8 days, or more extreme intervals of ≥ 28 days. (**c**) Heatmap representation of CTS gene classifier expression patterns in patients sub-grouped by hospital to ICU admission interval, that is, ≤ 1 day, ≥ 8 days, or ≥ 28 days. (**d**) Silhouette width analysis of patients sub-grouped by hospital to ICU admission interval. Positive subtype-specific and average widths indicate strong classifier performance. (**e**) Confusion matrices of CTS classified patients sub-grouped by hospital to ICU admission interval, that is, ≤ 1 day (CTS_1d), ≥ 8 days (CTS_8d), or ≥ 28 days (CTS_28d) against original CTS subtypes.

**Extended Data Fig. 4:**
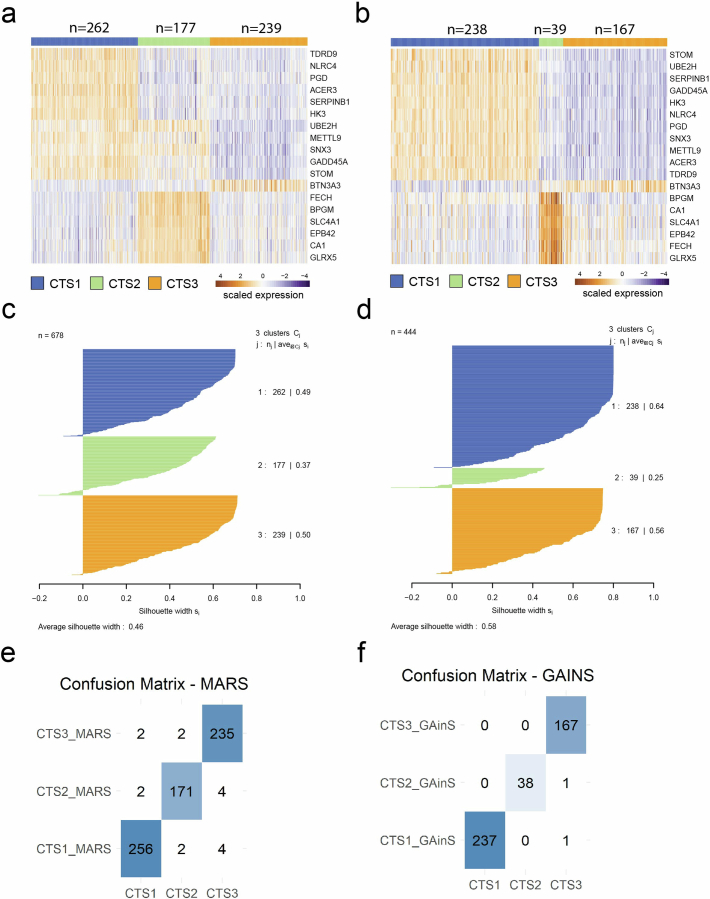
Classification of sepsis patients across study sites and classifier sensitivity. Heatmaps show classifier gene expression for patients assigned to CTSs in the MARS cohort (**a**) and GAinS cohort (**b**). Silhouette plots depict high classification quality in the MARS (**c**) and GAinS (**d**) cohorts. Confusion matrices compare original CTSs to CTS assignments from the gene classifier in the MARS (**e**) and GAinS (**f**) cohorts.

To enhance the robustness and generalizability of the CTS1–3 model, we first performed de novo consensus clustering on all samples obtained on ICU admission and unique genes (*n* = 7,260), as described previously^9^. This analysis identified an optimal cluster (subtype) size of three, reinforcing our CTS1–3 model (Extended Data Fig. 2a–e). Strong concordance was observed between CTS1 and CTS2, and de novo clusters 1 and 2, respectively, with 85.4% and 94.0% alignment (Extended Data Fig. 2f). The high concordance for CTS1 and CTS2 indicates that these subtypes were well captured by unsupervised clustering. In contrast, CTS3 showed mixed concordance with de novo cluster 2 (69.7%) and almost exclusively cluster 3 (29.3%; Extended Data Fig. 2f). This observation may reflect a transitional or mixed immune state, or limitations of the de novo clustering approach in capturing the distinct features of CTS3. Second, we tested our CTS gene classifier using publicly available RNA-seq data from a prospective, observational cohort of adults (≥18 years) hospitalized with severe, undifferentiated infection (suspected sepsis) at the Entebbe General Referral Hospital in Uganda (RESERVE-U)^25^. Our CTS gene classifier and random forest method on co-normalized RNA-seq data (*n* = 128) successfully assigned RESERVE-U patients to CTS1–3, providing evidence of demographic and geographical generalizability (Extended Data Fig. 2g,h). Additionally, we tested the sensitivity of the CTS gene classifier in the context of differing time intervals between hospital and ICU admission, which may represent a good proxy to the progression to organ dysfunction that triggers ICU admission. In our cohort, the median interval was 3 days (Extended Data Fig. 3a). Of the patients, 348 were admitted to the ICU for 1 day or less (first quartile, Q1) of hospital admission, 284 patients were admitted for 8 or more days (third quartile, Q3) and 44 patients were transferred to the ICU after 28 or more days. No substantial differences in CTS distribution were observed across the three admission groups (Extended Data Fig. 3b). The original CTS assignments overlapped almost completely with CTS classifications based on groups with different hospital-to-ICU admission intervals (98%, 99% and 98% for admission intervals of 1 day or less, 8 or more days and 28 or more days, respectively). Heatmap representations of classifier genes and CTS assignments across different ICU admission intervals are shown in Extended Data Fig. 3c. Silhouette and confusion matrix analyses further demonstrated the robust performance of the gene classifier across different ICU admission times (Extended Data Fig. 3d,e). Furthermore, classification of patients stratified according to cohort successfully identified CTSs, with positive silhouette widths and confusion matrix analyses supporting the robust performance of the gene classifier (Extended Data Fig. 4a–f). Based on these sensitivity analyses, we conclude that CTS gene classifier performance is unaffected by either the time interval between hospitalization and ICU admission or the study site.

**Extended Data Fig. 5:**
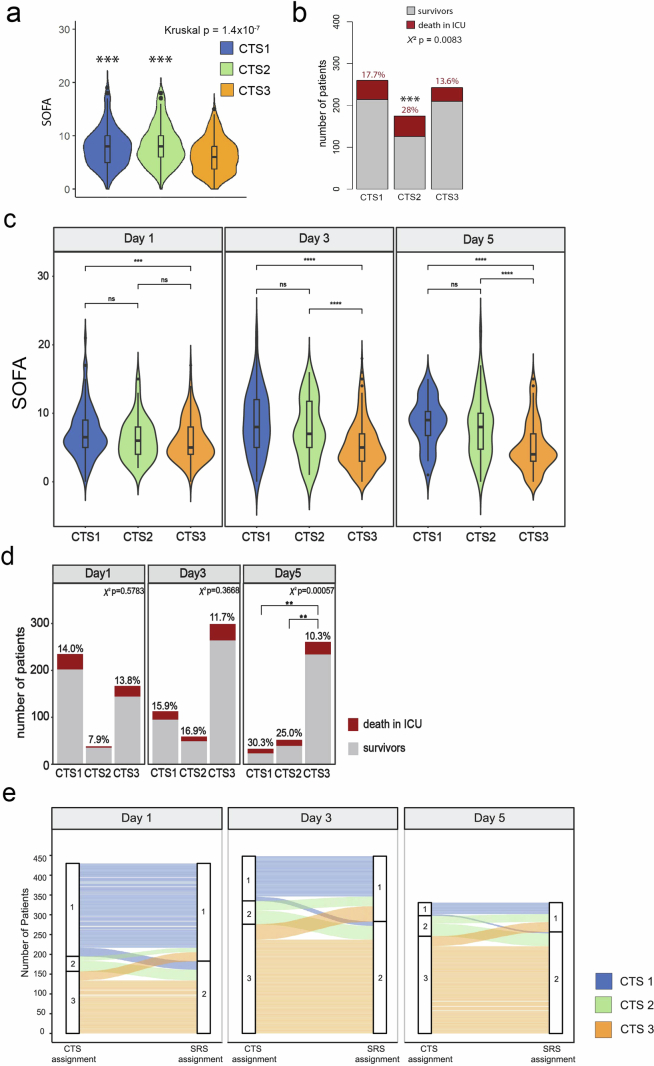
Assessment of clinical severity, outcome and longitudinal aspects against Consensus Transcriptomic Subtypes (CTS). (**a**, **b**) MARS patients (n = 678) classified into CTS 1-3, showing relationship to (**a**) Sequential Organ Failure Assessment (SOFA) scores (violin plot), and (**b**) ICU mortality (bar plot with percentages denoting patients assigned to each CTS who died in the ICU). Pairwise comparisons of SOFA scores between CTS groups were conducted using Dunn’s test, while ICU mortality was compared using pairwise Chi-squared tests with a Bonferroni correction. Dunn’s or Chi-squared post-hoc test p-value, * < 0.05, ** < 0.01, *** < 0.001 relative to CTS3. (**c**) SOFA scores of GAinS patients, stratified by CTS 1-3 on ICU days 1 (n = 444), 3 (n = 470), and 5 (n = 374). Data are shown as box and whisker plots, with pairwise comparisons performed using the Wilcoxon rank-sum test (two-sided). (**d**) ICU mortality rates corresponding to each CTS classification. Bar plots show the percentage of non-survivors within each group, analyzed by pairwise Chi-squared tests with Bonferroni correction. (**e**) Alluvial plots illustrating the dynamic classification of patients between CTS 1-3 and SRS1-2 across ICU days 1, 3, and 5. For all relevant panels, asterisks denote statistical significance: *p < 0.05, **p < 0.01, ***p < 0.001, ****p < 0.0001, and “ns”, not significant. Violin plots (**a**, **c**) illustrate the distribution of SOFA scores from individual patients (the unit of study), stratified by CTS, where the central line marks the median, the box defines the interquartile range (IQR), and whiskers extend to 1.5 times the IQR.

Classifying patient samples as CTS1–3 was substantially associated with the sequential organ failure assessment (SOFA) score (Fig. 1e), acute physiology and chronic health evaluation IV (APACHE IV) score (Fig. 1f) and ICU mortality (Fig. 1g). CTS1-classified and CTS2-classified patients were relatively more severe, with CTS2 patients having the highest APACHE IV scores and rate of deaths in the ICU. Evaluating the association of CTS assignment on admission with the SOFA score in MARS and GAinS patients revealed consistent relationships (Extended Data Fig. 5a,c). The analysis of CTS membership in relation to ICU mortality among MARS and GAinS patients indicated distinct outcomes with a substantial association observed solely in MARS patients classified according to CTS (Extended Data Fig. 5b,d). CTS assignment was associated with the primary anatomical site of infection, where a higher percentage of CTS1-classified patients were diagnosed with intra-abdominal infection or fecal peritonitis (44.4%), while 69.2% of CTS3 patients were diagnosed with pneumonia (Table 1). Next, using Levene’s test for homogeneity of variance in APACHE IV scores across CTSs resulted in a nonsignificant outcome (*P* = 0.143), suggesting similar variance across CTSs. Furthermore, we assessed the ability of APACHE IV scores to classify patients into CTS1–3 using ROC curves and evaluated net reclassification improvement (NRI) to determine whether CTS adds predictive value beyond APACHE IV in mortality risk assessment. ROC analysis yielded AUC values of 0.56, 0.66 and 0.54 for CTS1, CTS2 and CTS3, respectively, indicating poor discrimination of CTS subtypes by APACHE IV scores. Reclassification tables showed minimal shifts, with only six of 241 non-survivors and four of 881 survivors being reclassified. Bootstrap NRI estimates yielded narrow confidence intervals (0.0128–0.0172), suggesting marginal improvement in classification performance. These findings indicate that while APACHE IV captures overall illness severity, it does not effectively distinguish CTS subtypes, reinforcing the notion that CTS represents a distinct dimension of patient heterogeneity not captured by conventional clinical severity scores. To explore patient trajectories within assigned CTSs throughout their ICU stay, we classified blood transcriptomic data from the GAinS cohort, collected on days 3 and 5 after ICU admission (*n* = 817), to CTS1–3 using the 18-gene CTS classifier, and analyzed these together with the ICU admission samples (*n* = 444). We found that CTS patterns were dynamic over the ICU stay, as illustrated by trajectories from 35 patients with blood transcriptomic data available at days 1, 3 and 5 (Fig. 1h). Patients who remained in CTS1 and CTS2 at later time points had more severe disease and worse outcomes (Extended Data Fig. 5b,d), which is consistent with differences in disease progression and response to therapy according to CTS group. The proportion of patient clustering between CTS and SRS classifiers was lower at later time points (Extended Data Fig. 5e).

### CTSs and the impact of corticosteroid use

**Fig. 2:**
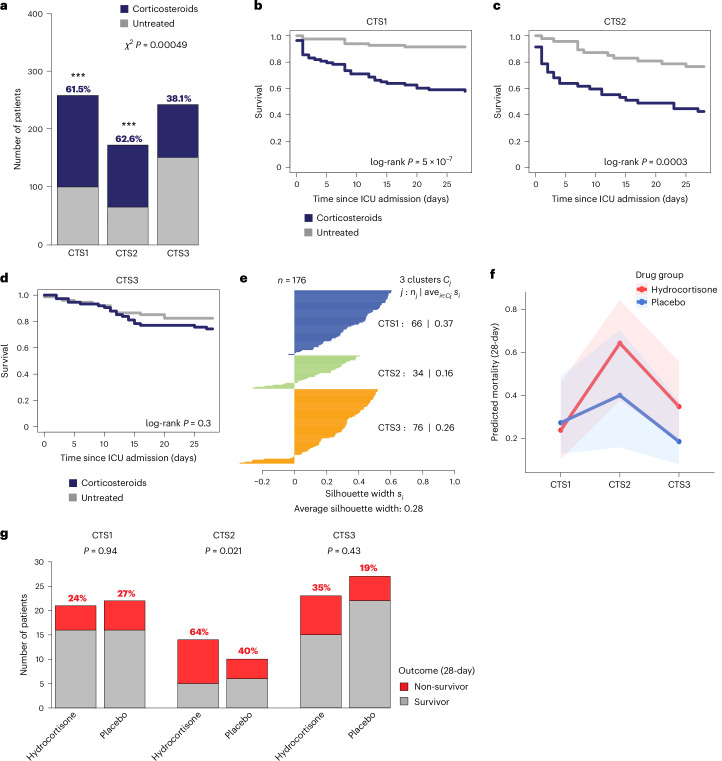
Evaluation of the impact of corticosteroid treatment on the 28-day mortality rates of patients classified as CTSs. **a**, Bar plot depicting corticosteroid-treated and untreated patients in the MARS cohort (*n* = 617) assigned to CTS1, CTS2 or CTS3, and analyzed using pairwise chi-squared tests with Bonferroni correction. **b**–**d**, Kaplan–Meier survival analysis of corticosteroid-treated or untreated subgroups in CTS1-assigned (**b**), CTS2-assigned (**c**) and CTS3-assigned (**d**) MARS patients. **e**, Silhouette width analysis of the 176 unique patient samples from the VANISH randomized clinical trial assigned to CTS1, CTS2 or CTS3, illustrating overall model fit and cluster stability. *C*_*j*_ is cluster index (*j* = 1, 2, 3); *n*_*j*_ denotes number of observations (points) in cluster *j*; ave_*i∈C*__*j*_ denotes the mean silhouette width of all points in that cluster. **f**, Line plot depicting predicted 28-day mortality as the outcome variable in hydrocortisone-treated or untreated patients stratified into CTS1–3. The lines indicate the mean predicted mortality; the shaded areas denote the 95% CIs. **g**, Bar plots of VANISH study patients stratified into CTS1–3 illustrating the main effect outcome (28-day mortality) with and without hydrocortisone treatment. A chi-squared post hoc test was used. ***P < 0.01 relative to CTS3.

To test the effect of corticosteroid treatment on outcome (mortality) across CTSs, we evaluated the number of patients treated in the MARS cohort (treated *n* = 362, non-treated *n* = 316). A chi-squared test of the association between corticosteroid treatment and CTS assignment revealed a substantial relationship. Specifically, 61.5% and 62.5% of patients assigned to CTS1 and CTS2, respectively, were treated with corticosteroids on ICU admission, compared to 38.1% in CTS3 (Fig. 2a). To further explore the impact of corticosteroid treatment on mortality across the different CTS assignments, we used propensity score matching. This method allowed for the balancing of covariates between treated and untreated patients, ensuring that the treatment groups were comparable. By estimating the probability of receiving corticosteroid treatment based on age, SOFA score, primary site of infection, hospital-to-ICU admission intervals and septic shock diagnosis, we matched 408 patients (Table 2). Following this, patients assigned to CTS1, CTS2 or CTS3 were largely similar in baseline characteristics, with minor differences observed in the diagnosis of abdominal sepsis, pneumonia and hospital-to-ICU time interval. Next, we fitted a binomial logistic regression model to assess the effect of corticosteroid use on 28-day mortality across CTS1, CTS2 and CTS3. Corticosteroid use significantly increased the odds of 28-day mortality (odds ratio (OR) = 3.83, 95% confidence interval (CI) = 2.38 to 6.28, *P* = 5.2 × 10^−8^). Without corticosteroid use, matched CTS1-classified patients had the lowest baseline mortality (OR = 0.15, 95% CI = 0.09 to 0.25, *P* = 5.6 × 10^−14^), followed by CTS3 (OR = 0.80, 95% CI = 0.46 to 1.38, *P* = 0.42) and CTS2 (OR = 2.14, 95% CI = 1.22 to 3.8, *P* = 0.00085). The latter suggests that CTS2 membership is associated with a significantly higher risk of mortality up to 28 days. The interaction term for CTS2-assigned patients showed no significant alteration in the corticosteroid effect compared to CTS1 (OR = 0.56, 95% CI = 0.16 to 1.94, *P* = 0.363). The interaction term in CTS3 was significant (OR = 0.2, 95% CI = 0.06 to 0.66, *P* = 0.009), indicating that corticosteroid use in CTS3 patients was associated with a significant reduction in mortality compared to the reference group (CTS1). This suggests that corticosteroids may have a protective effect for patients assigned to CTS3 relative to CTS1. Overall, the model explained a substantial portion of the variation, with an Akaike information criterion of 441.38, indicating a good fit. Kaplan–Meier survival curves and log-rank *P* values are illustrated in Fig. 2b–d. Substantially, the 28-day mortality rate among patients assigned to CTS1 and CTS2 who received corticosteroid treatment increased by 33.7% and 34%, respectively, relative to their untreated counterparts, with log-rank *P* values equating to 5 × 10^−7^ and 0.0003 (Fig. 2b,c). Next, we evaluated the blood transcriptomes of patients enrolled in the vasopressin vs noradrenaline as initial therapy in septic shock (VANISH) clinical trial^13,26^. VANISH investigators compared norepinephrine to vasopressin with or without hydrocortisone for the treatment of septic shock. A total of 176 patients were classified as CTS1–3 (Fig. 2e). A logistic regression model assessing hydrocortisone treatment on 28-day mortality with interaction terms showed higher predicted probabilities of 28-day mortality in hydrocortisone-treated CTS2-classified and CTS3-classified patients (Fig. 2f). The interaction terms were not significant, suggesting no strong evidence that the effect of the drug differed across CTS subtypes. In line with our previous observations (Fig. 1f), the main effect of CTS2 was significant (OR = 5.76, 95% CI = 1.31 to 25.4, *P* = 0.021), indicating that it increased the odds of mortality relative to CTS1 and CTS3 (Fig. 2g).

**Table 2:** Clinical characteristics of propensity-score-matched MARS patients classified as CTS1–3

	CTS1	CTS2	CTS3	*P*
Patients, *n*	166	94	148	
Demographics				
Age, mean (s.d.)	60.58 (16.18)	60.99 (13.09)	61.43 (14.14)	0.878^a^
Sex, males (%)	96 (57.8)	52 (55.3)	87 (58.8)	0.866^b^
Site of infection, *n* (%)				
Lung	73 (44.0)	41 (43.6)	97 (65.5)	0.001^b^
Abdominal	61 (36.7)	28 (29.8)	9 (6.1)	0.001^b^
Severity of disease, median (Q1–Q3)				
APACHE IV score	80.00 (64.00–95.75)	85.00 (64.25–111.75)	79.50 (64.75–93.50)	0.106^c^
SOFA score	8 (5–10)	8 (5–10)	7 (5–9)	0.077 ^c^
Complications				
Septic shock, *n* (%)	66 (39.8)	38 (40.4)	43 (29.1)	0.086 ^b^
Other, median (Q1–Q3)				
Hospital-to-ICU admission interval	1 (0–4)	2 (0–9)	1 (0–5)	0.031^c^
Length of ICU stay, days	6 (2–14)	8 (2–12)	7.5 (4–12)	0.31^c^

^a^Two-sided ANOVA. ^b^Chi-squared test after Bonferroni correction. ^c^Kruksal–Wallis test.

### Characterization of biological pathways and single-cell patterns of the CTSs

**Fig. 3:**
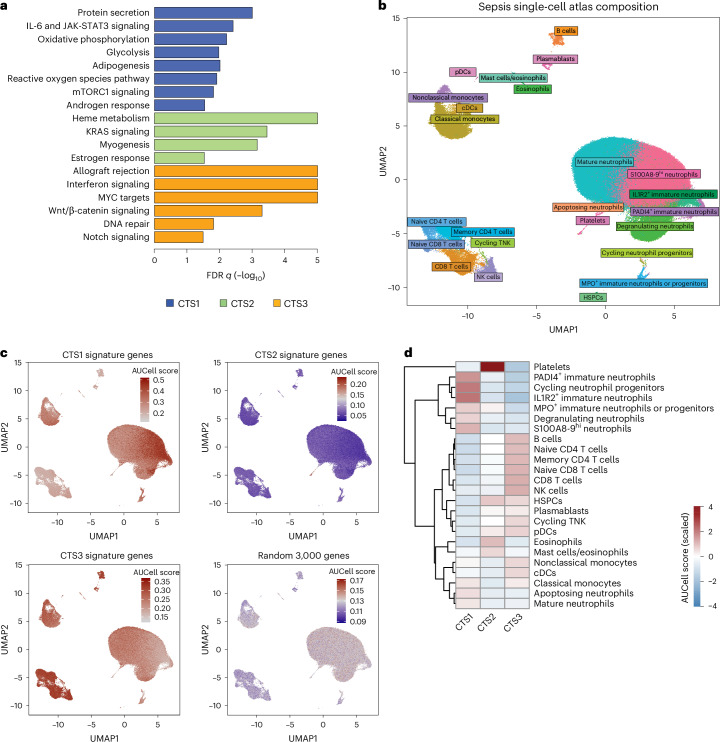
Characterization of biological pathways and cell-type-specific patterns of the CTSs. **a**, GSEA of CTSs using the ‘hallmarks’ molecular signatures database. **b**, Dimensionality reduction of scRNA-seq (scdata using uniform manifold approximation and projection (UMAP) and cell-type-specific annotations in the single-cell atlas^27^). **c**, Projection of the AUCell-determined CTS scores to the single-cell atlas UMAP and differential gene expression levels. **d**, Heatmap representation of AUCell scores per cell population in the sepsis single-cell atlas. cDC, conventional dendritic cell; FDR, false discovery rate; MPO, myeloperoxidase; pDC, plasmacytoid dendritic cell; TNK, thymic natural killer cells.

**Extended Data Fig. 6:**
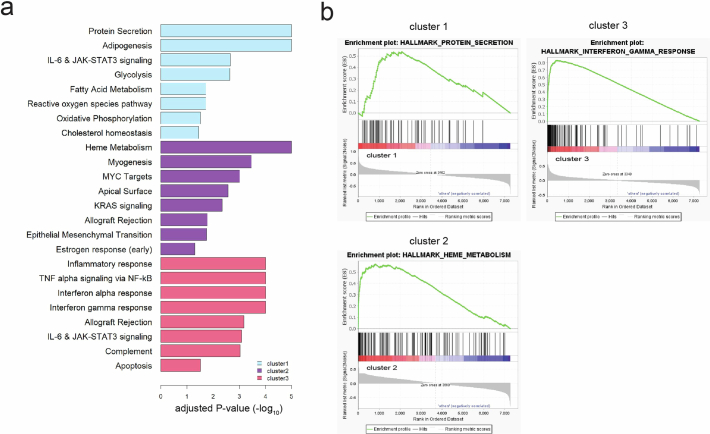
Biological characterization of *de novo* clusters 1-3. (**a**) Barplots depicting significant enrichment (adjusted p-value < 0.05) of gene sets per de novo cluster based on Gene Set Enrichment Analysis (GSEA) “hallmarks”. (**b**) Enrichment plots derived from GSEA of top “hallmarks” per de novo cluster.

**Extended Data Fig. 7:**
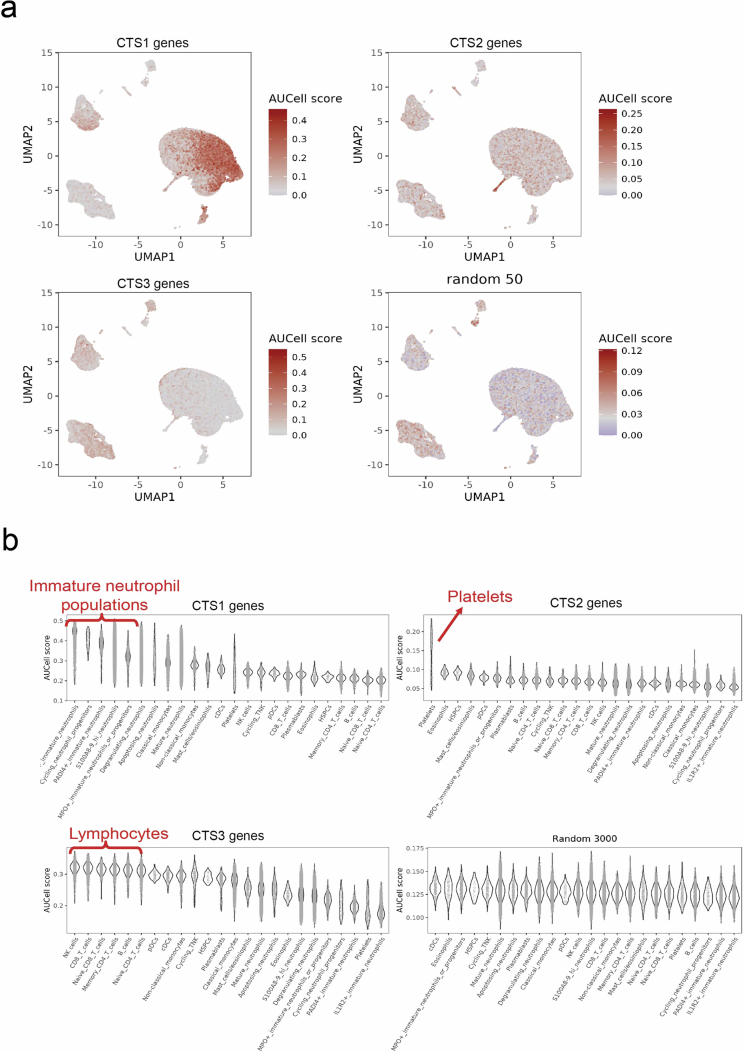
Single-cell patterns of the consensus transcriptomic subtypes. (**a**) AUCell scores of each cell in the single-cell atlas (ref. ^27^; Kwok et al) based on CTS1, CTS2, and CTS3-specific gene sets (30 genes per CTS) projected to Uniform Manifold Approximation and Projection (UMAP) (**b**) Violin plots depicting cell type-specific distribution of CTS scores.

To delineate the biological characteristics of each CTS at ICU admission, we investigated additional molecular data available from a subset of patients, and gene set enrichment analysis (GSEA) and single-cell RNA-seq (scRNA-seq) data mining. GSEA revealed differential abundance of distinct gene expression pathways per CTS, thus providing substantial insight into the putative biological underpinnings of the subtypes (Fig. 3a). CTS1 was characterized by typical pro-inflammatory pathways that included interleukin-6 (IL-6) signaling, reactive oxygen species pathway, glycolysis, adipogenesis, oxidative phosphorylation and mammalian target of rapamycin complex 1 (mTORC1) signaling. CTS2 was robustly associated with a heme metabolism pathway, KRAS signaling, myogenesis and estrogen response pathways, whereas allograft rejection, Wnt β-catenin, MYC, interferon signaling and DNA repair pathways characterized CTS3 (Fig. 3a). Transcript-level concordance between CTSs and de novo clusters was evaluated using GSEA, identifying multiple gene sets in de novo clusters 1, 2 and 3 that distinguish CTS1, CTS2 and CTS3 (Extended Data Fig. 6a,b). Furthermore, the GSEA results showed that while de novo cluster 3 was enriched for the interferon signaling and allograft rejection pathways similar to CTS3, there was overlap with other pathways characterizing CTSs, suggesting that the mixed concordance of CTS3 may be due to overlapping biology that could not be resolved by de novo clustering. Next, we investigated the putative cell-type specificity of the CTSs using a recently generated single-cell map of whole blood in sepsis^27^. The composition and cellular annotations of the sepsis single-cell atlas are illustrated in Fig. 3b. Briefly, we used AUCell^28^ to score each cell in the single-cell atlas based on CTS1-specific, CTS2-specific and CTS3-specific gene sets (30 genes per CTS). This analysis showed that the CTS1 and CTS2 gene sets scored high in immature neutrophils and platelets, respectively (Extended Data Fig. 7a). CTS3 exhibited moderately increased scores in lymphocytes. This analysis was then repeated using a larger gene set. Namely, gene expression levels were compared between samples in each CTS and differentially expressed genes were filtered to retain only those genes uniquely upregulated in each CTS (~3,000 genes per set). Next, these gene sets were used to perform cell scoring. The results from this test reinforce our previous analysis where CTS1 genes were upregulated in immature neutrophils, including protein-arginine deiminase type-4 (PADI4)^+^ and interleukin-1 receptor type 2 (IL1R2)^+^ neutrophils, while CTS2 genes were upregulated in platelets and eosinophils (Fig. 3c,d). CTS3 genes may be upregulated across all lymphocyte populations (B, T and natural killer (NK) cells), and nonclassical monocytes. Cell-type-specific distribution of CTS scores corroborates these findings (Extended Data Fig. 7b).

### CTSs and host response biomarkers

**Fig. 4:**
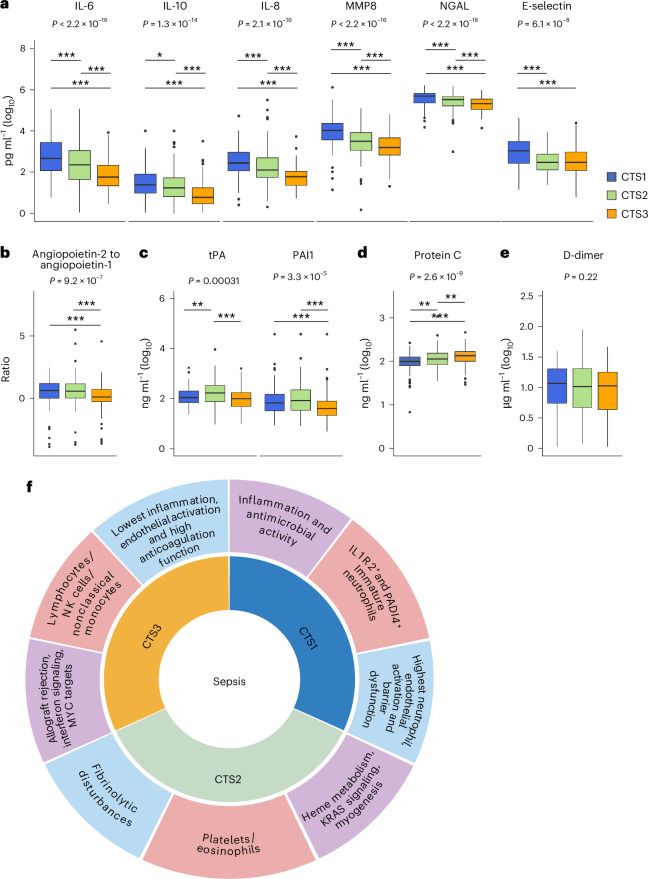
Host response biomarker profiles across CTSs of sepsis. **a**–**e**, Box plots depicting the levels of soluble mediators of inflammation and endothelial function (**a**), endothelial barrier integrity (**b**), fibrinolysis (**c**), anticoagulation (**d**) and clot breakdown (**e**), measured in CTS1-classified (*n* = 157), CTS2-classified (*n* = 138) and CTS3-classified (*n* = 121) patients from the MARS cohort, and analyzed using a Dunn’s post hoc test. Kruskal–Wallis exact *P* values are reported. Dunn’s post hoc test: *P < 0.05, **P < 0.01, ***P < 0.001 relative to CTS3. The box plots illustrate the distribution of each biomarker from individual patients (the unit of the study), stratified according to CTS, where the central line marks the median, the box defines the interquartile range and the whiskers extend to 1.5 times the interquartile range. **f**, Proposed taxonomy of the CTSs of sepsis, reflecting cell types that probably drive each CTS endotype (light red slices), the top-most significant cellular biological pathways (purple slices) and the host response biomarker profiles (light blue slices). Graphic in **f** created using BioRender.com.

To obtain further insight into the pathobiological features of CTSs, we analyzed a set of plasma biomarkers reflective of changes in key pathophysiological domains implicated in sepsis measured in the MARS cohort. CTS1-classified patients had the highest levels of cytokine and neutrophil activation markers, including IL-6, IL-10, IL-8, matrix metalloproteinase-8 (MMP8) and neutrophil gelatinase-associated lipocalin (NGAL) (Fig. 4a). In addition, high levels of soluble E-selectin and angiopoietin-2-to-angiopoietin-1 ratio (reflective of endothelial activation and disturbed barrier function, respectively) were observed in CTS1 (Fig. 4a,b). Higher levels of the fibrinolysis markers tissue-type plasminogen activator (tPA) and plasminogen activator inhibitor type 1 (PAI1) were found for patients classified as poor-prognosis CTS2 (Fig. 4c). Protein C levels were significantly different between CTSs, with higher levels found in CTS3 and lowest in CTS1-classified patients (Fig. 4d). D-dimer levels were not different between groups (Fig. 4e), suggesting that although there may be differences in fibrinolysis and anticoagulation, the overall rate of clot formation and breakdown was consistent across subtypes. Altogether, these findings indicate that CTS1 has features of an increased inflammatory state and neutrophil subsets and function. CTS2 was characterized by a heme metabolism transcriptional signature and fibrinolytic disturbances relative to other subtypes. CTS3-classified patients were consistent with less severe cellular and molecular perturbations.

## Discussion

This study represents a collaborative effort by a group of investigators who earlier independently published transcriptomic sepsis subtypes to establish a standardized blood transcriptomics-based strategy to classify patients with all-cause sepsis. Through cooperative bioinformatics work on a large collection of patients with sepsis from two cohorts, we developed a consensus blood transcriptomic classification strategy that can categorize critically ill patients with sepsis into three distinct molecular subtypes with pathophysiological implications. The proposed taxonomy is supported by substantial variations in the underlying biological characteristics of each subtype, particularly CTS1 and CTS2 (Fig. 4f). Thus, we have improved our understanding of the sepsis transcriptomic subtypes and exposed previously unknown associations between heme metabolism and coagulation underlying CTS2-classified patients, which make up approximately 20% of patients with sepsis studied. We propose that the adoption of this new taxonomy, consisting of CTS1 (IL1R2^+^ and PADI4^+^ immature neutrophils; inflammatory and endothelial), CTS2 (platelets and eosinophils; heme metabolism and coagulation) and CTS3 (lymphocytes, nonclassical monocytes and NK cells; interferon signaling, Wnt/β-catenin, DNA repair), will enhance future research involving the field of sepsis molecular subtypes and can be adopted by the scientific community for the purpose of resolving sepsis heterogeneity by categorizing the systemic host response.

From a clinical standpoint, it is yet uncertain which characteristics will serve as the most pertinent for the subclassification of patients with sepsis. Several studies have sought to subclassify patients with sepsis using diverse data sources, including clinical parameters and molecular data^29,30^. Members of this study group and others^22^ have also compared sepsis subclassification strategies in one cohort, which illustrated little concordance between molecular and clinical subphenotypes^31^. It is unlikely that one strategy will outperform others in reliably assigning patients with sepsis to subtypes or subphenotypes. Instead, a combination of factors representing dominant facets of sepsis pathophysiology is more likely to resolve sepsis heterogeneity. However, it is uncertain whether a mixture of clinical parameters and molecular profiles is necessary for accurately identifying treatable traits and predicting therapeutic responses. It is crucial to highlight that while CTSs show higher levels of certain soluble mediators of the host response, for example, pro-inflammatory cytokines for CTS1 and coagulation markers for CTS2, these associations alone do not enable classification into blood transcriptomic subtypes. This emphasizes the concept that transcriptional signatures may provide a more precise tool to classify diseases compared to currently advocated biomarkers or clinical measurements^32^. These signatures provide valuable information on the mechanisms of diseases^33^, assist in the process of diagnosis^34–36^ and may be used to inform therapy decisions^13^. Moreover, transcriptional markers have had a crucial role in revealing the diversity of disorders, such as autoimmune diseases. The use of immune cell transcriptional profiling has improved our capacity to identify changes in disease activity and make well-informed decisions on treatment^37^. These signatures have also been used to predict the future course of autoimmune disorders, indicating their importance in assessing the seriousness of the condition and the likelihood of relapse^38^. Furthermore, our results support the application of CTSs for tracking patient trajectories throughout their ICU stay. Given that we were limited to 35 patients having samples at all three time points, we acknowledge that observations regarding CTS dynamics remain exploratory. Dynamic assessment of CTSs may support adaptive clinical management in sepsis by enabling real-time stratification of immune status, informing immunomodulatory treatment decisions and monitoring response to therapy. CTS classifiers were derived from ICU admission samples, which may be a factor in the reduced concordance between CTS and SRS clustering observed with day 3 and day 5 samples, and highlights the need for additional work to address this and more fully understand how rapidly sepsis subtypes shift at the individual patient level. Although exploratory, our findings highlight the potential value of incorporating serial CTS profiling into future clinical trials and precision-guided treatment strategies in sepsis.

We describe an 18-gene classifier that enables CTS classification with a high accuracy for ICU admission. Recent advancements in molecular diagnostics have addressed the concerns raised about rapid gene expression analysis in critical care settings. Tests like TriVerity, Biomeme HR-B/V and BioFire HR-B/V analyze 22–42 genes with turnaround times of 30–60 min, demonstrating the feasibility of implementing such systems in routine clinical care^39,40^. While molecular tests probably will come with relatively high costs, their potential to improve patient outcomes and reduce overall healthcare expenses warrants cost-effectiveness research in specific clinical contexts.

Depicting sepsis in a quantitative and clinically relevant way requires a substantial investment of effort and resources. We posit that by identifying molecularly homogeneous subgroups of patients with sepsis and characterizing putative ‘driver’ events in these samples, we might enhance the development of specific therapies. Recently, members of this research group observed a substantial rise in the number and heterogeneity of neutrophils in sepsis, indicating an increased production and release of immature granulocytes into the bloodstream during emergency granulopoiesis, which was significantly enriched in patients with the SRS1 subtype^27^, with evidence of expansion of immature neutrophil populations (notably IL1R2^+^ cells) and immunosuppressive effects on T cells in vitro^27^. Single-cell multi-omic profiling of circulating hematopoietic stem and progenitor cells (HSPCs) showed epigenetic and transcriptomic signatures of altered emergency granulopoiesis and STAT3-mediated gene regulation^27^, while genetic associations with SRS further implicate CEBPB^21^, a master regulator of emergency granulopoiesis. Our findings also showed that CTS1 was associated with PADI4^+^ neutrophils, driving the formation of DNA-histone structures called neutrophil extracellular traps, which trap and kill pathogens but also promote endothelial injury and organ dysfunction^41–43^. Platelets and eosinophils emerged as prominent features of CTS2. While platelet involvement in sepsis is well documented^44^, the role of eosinophils remains less explored. Persistently low eosinophil numbers are associated with adverse clinical outcomes in sepsis, but their mechanistic contribution to immune dysregulation is not clear^45^. CTS3 was characterized by broad lymphocytic features, as well as nonclassical monocytes (CD14^lo^CD16^hi^). Nonclassical monocytes produce pro-inflammatory cytokines (for example, TNF, IL-12) upon stimulation, contributing to antimicrobial defense^46^. However, their role is context-dependent, balancing protective immunity with potential tissue-damaging inflammation. While still speculative, these observations offer the possibility of facilitating the development of new tailored treatments for sepsis, which would involve testing therapeutics in more homogenous patient subgroups^13^. In our study, pseudo-randomization of patients with all-cause sepsis from the MARS cohort (not solely septic shock) stratified according to CTSs, uncovered a harmful signal in CTS1 (inflammatory) and CTS2 (coagulation/fibrinolysis) patients who were treated with corticosteroids; a reanalysis of the VANISH trial suggested harm inflicted by steroid treatment in CTS2 patients. Despite our efforts to address potential confounders in the MARS observational cohort through propensity score matching and to retrospectively test the CTS classification method in a randomized controlled trial (RCT), the therapeutic implications of CTS classification require validation through well-designed prospective RCTs. The CTS2 subtype may be associated with corticosteroid resistance because of enhanced heme metabolism causing oxidative stress^47^, increased KRAS signaling disrupting glucocorticoid receptor function^48^ and augmented estrogen receptor function competing with glucocorticoid receptors for coactivators^49^. In addition, hydrocortisone may further enhance the coagulation features of the CTS2 subtype, that is, corticosteroids increased tissue factor expression, a key driver of coagulation in sepsis in monocytic cells^50^, while oral prednisolone enhanced coagulation activation in healthy individuals intravenously administered with endotoxin^51^. Our findings, taken together with an earlier study indicating harm associated with hydrocortisone treatment in the SRS2 subtype^13^, suggest potential far-reaching clinical implications of molecular classification strategies compared to traditional clinical observations and subphenotyping.

Clustering and subclassification strategies, even if based on what are important characteristics of the host response to sepsis, may not necessarily be able to accurately predict differential treatment responses. This may arise because of the acute medications themselves, with diverse modes of action affecting several cellular biological pathways simultaneously, or because of our limited ability to accurately determine pathway involvement or interactions using static ‘omics’ data. Similarly, prediction of outcomes, such as mortality, are of limited utility given the highly multifactorial nature of final cause of death in sepsis and the relationship with comorbidities. Cohort differences relating to management, case mix and unidentified factors are further potential confounders of such associations, as we have reported previously for SRS1 with MARS and GAinS^19^. Our current research intends to develop a comprehensive framework for systematic analysis of sepsis molecular heterogeneity in several clinical contexts, which is currently considered the most accurate description available. We anticipate that it will also expedite the implementation of CTS classifications to in vitro models that encompass primary immune cells, cell lines and organoids in the context of immune functional assays. Our findings demonstrate the robustness of the CTS gene classifier across independent cohorts. While sepsis biology is influenced by heterogeneous and region-specific factors, including genetics, epigenetics, resource availability and clinical thresholds for ICU admission, recent work by Moore et al.^52^ identified similar immune subtypes using transcriptomic, single-cell and protein data, reinforcing the broader applicability of these immune subtypes. Consistent with the findings by Moore et al.^52^, CTS1, CTS2 and CTS3 broadly correspond to clusters characterized by myeloid dysregulation, lymphoid dysregulation and a lymphoid protective response, respectively. Our results, and those from the companion study by Moore et. al.^52^ suggest potential cross-cohort generalizability but highlight the need for additional multi-omics validation across diverse populations, particularly low- to middle-income regions, and for study over time, to understand the relationship to the natural history of the acute illness. Nonetheless, while our study underscores distinct biological characteristics among subtypes, it is limited by the fact that the observed associations are correlational in nature and focused on informativeness for ICU admission. As such, and inherent to the observational nature of this investigation, these findings cannot be used to draw conclusions about cause-and-effect relationships.

In conclusion, we believe that the standardized framework outlined in this study offers a shared basis to resolve sepsis molecular heterogeneity in the clinical context. It is expected to undergo further iterations and enhancements in parallel with technological advancements that will enable incorporation of more data sources. We envisage that this will improve our understanding of sepsis pathophysiology and provide a key bridge to establish precision medicine approaches in the context of critical care.

## Methods

### Study design

The overall design and analytical workflow of this study are described in Fig. 1a. There were three participating research groups, each of which had previously developed and published an approach for classifying patients with sepsis as transcriptomic subtypes using whole-blood samples^8–11^. This study focused on the secondary analysis of existing de-identified transcriptomic, soluble biomarker and clinical data. No personal identifiable information was included in these datasets. Each dataset was processed and co-normalized (see section on ‘RNA transcriptomic data preprocessing and co-normalization’). Although this decision precluded an analysis of the impact of gene expression normalization on subtype assignments, it allowed this study to focus on biological interpretations and evaluate the clinical relevance of the different subtypes versus bioinformatic approaches. Each expert group then applied their classification strategy to the datasets in the common aggregated dataset.

### Ethics

This study was conducted in accordance with the principles outlined in the Declaration of Helsinki (October 2013) and the ethical standards of the committees responsible. MARS patients (ClinicalTrials.gov registration no. NCT01905033) were included via an opt-out consent method approved by the institutional review boards (IRBs) of both recruiting hospitals, that is, Amsterdam UMC and UMC Utrecht (IRB no. 10-056C). For UK GAinS, ethical approval was granted nationally and locally, with written informed consent obtained from all patients or their legal representative and conducted under Research Ethics Committee approval nos. 05/MRE00/38, 08/H0505/78 and 06/Q1605/55.

### Leukocyte RNA isolation and bioinformatics

For the MARS cohort, total RNA (RNA integrity number > 6.0; Agilent Bioanalyzer) was isolated using the PAXgene Blood RNA isolation kits (QIAGEN) to generate complementary DNA (cDNA) for hybridization to the human genome U219-96 array plate (Affymetrix) at the Cologne Center for Genomics. Preprocessing and quality control were performed using the affy method (v.1.36.1)^53^. Array data were background-corrected using robust multi-array average, quantile-normalized and summarizedusing median polish. The occurrence of nonexperimental chip effects was evaluated using the surrogate variable analysis (SVA) method (v.3.4.0) and corrected using the empirical Bayes method ComBat^54^. In a second population of patients from the MARS cohort (*n* = 156), cDNA was hybridized to Affymetrix Human Transcriptome Array 2.0 (Thermo Fisher Scientific) as described previously^20^. After robust multi-array average background-correction, quantile normalization and log_2_-transformation using the oligo method (v.1.44)^55^, data were evaluated for nonexperimental chip effects using SVA (v.3.28) and adjusted using the ComBat method. Probes were annotated using biomart (v.2.36.1); low expression probes were filtered using the genefilter method (v.1.62).

For the GAinS cohort, sample collection was performed as described in refs. ^8^ and ^19^. Briefly, whole blood (~10 ml) was obtained from patients with sepsis on the first, third and/or fifth day after ICU admission. Leukocyte isolation was performed using the LeukoLOCK system (Thermo Fisher Scientific), with total RNA extracted using the Total RNA Isolation Protocol (Ambion). Samples were either hybridized to Illumina HumanHT-12 V4.0 expression beadchips or used for RNA-seq. Regarding the latter, cDNA libraries were prepared using the NEB Ultra II LIbrary Prep kits (Illumina) and sequenced using a NovaSeq 6000 system (Illumina). Reads were aligned to the reference genome (GRCh38 v.99) using STAR (v.2.7.3) and quantified using featureCounts^55,56^. Counts were then normalized and log-transformed, resulting in log-counts per million. Raw microarray data were processed using GenomeStudio.

### RNA transcriptomic data preprocessing and co-normalization

**Extended Data Fig. 8:**
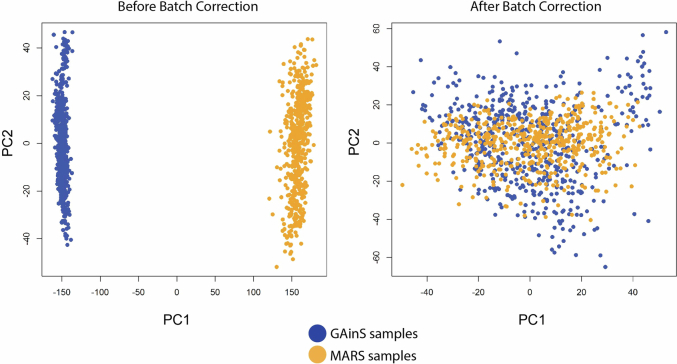
Principal component analysis before and after batch correction. Dotplots illustrating the blood transcriptomes of the Genomics Advances in Sepsis (GAinS) and Molecular Diagnostics and Risk Stratification of Sepsis (MARS) cohorts, before and after batch correction.

All datasets were combined by first remapping the microarray oligonucleotide probe sequences provided by Affymetrix and Illumina to GRCh38.p7 using Bowtie2 (ref. ^57^) as described previously^58^. Sequences labeled as ‘perfect match’ were annotated using Biomart^59^. We selected protein-coding biotypes for further analysis. The datasets were subsequently merged according to transcript name using Ensembl transcript IDs. Normalized data of the microarray and RNA-seq studies were then adjusted for nonexperimental batch effects using the empirical Bayesian method ComBat available in the SVA package (‘sva’) in R^54^. Specifically, gene expression was modeled as a function of platform differences (batches), with batches treated as categorical covariates. A vector indicating batch assignment (MARS-U219affy, MARS-HTA2.0affy, GAinS-Illumina, GAinS-RNA-seq, RESERVEU-RNA-seq or VANISH-Illumina) was created for each sample. Batch-associated variation was estimated and corrected using the parametric empirical Bayes framework implemented in ComBat (sva package). Transcripts were collapsed to unique genes by calculating the mean expression of transcripts from the same gene locus, which resulted in 7,260 unique gene expression values. Data were inspected before and after co-normalization using principal component analysis (Extended Data Fig. 8).

### Classification of sepsis transcriptomic subtypes specific to research groups

For GSE65682 (ref. ^9^) and GSE134347 (ref. ^20^) Mars1–4, a previously described 140-gene signature was used to train a random forest classifier and assign patient samples. For E-MTAB-4421, E-MTAB-4451 and EGAD00001008730 (refs. ^8,21^) SRS 1 and 2, patient samples were assigned to SRS1 or SRS2 using the SepstratifieR seven-gene method as described previously^19^. The Stanford inflammapathic, adaptive and coagulopathic labels were assigned based on the geometric means of expression levels of 33 genes^11^.

### Network analysis

To evaluate the association between the three classification systems (total number of subtypes equating to nine), we used a network-based approach. The association was calculated on the basis of Jaccard similarity coefficients and bootstrapped probabilities, defined as the size of the intersection between two sample sets over the size of their union, using the jaccard package in R. To quantify the statistical significance of subtype associations, we performed hypergeometric tests for overrepresentation of samples classified to one subtype in another. The resulting *P* values were adjusted for multiple hypotheses testing using the Benjamini–Hochberg method^24^. Next, to identify CTSs, we used a consensus clustering approach involving partitioning of the network into clusters using the Markov clustering available in the MCL package in R^60^. MCL is a scalable and efficient unsupervised clustering algorithm for networks. We evaluated clustering performance using silhouette widths using the R package cluster. Importantly, network granularity was controlled using a standard inflation factor, which we set at two, after testing inflation factors 1–7 (beyond inflation factor seven was flagged as erroneous). After setting the inflation factor at two, the MCL method was repeated ten times to ascertain the robustness and stability of clusters.

### Derivation of a CTS classifier

To derive a sepsis CTS classifier, we first selected patient samples from ICU admissions labeled as ‘core’ samples based on correspondence between the initially assigned subtype (MARS, GAinS or Stanford) and the subtype associated with each CTS. Subsequently, the top 5,000 genes were ranked according to nonparametric significance using a Kruskal–Wallis test using a one-versus-all scheme. Using a random forest classifier available in the CMA R package (supervised classification with high dimensional data methods)^61^, we assessed CTS classification with tenfold cross-validation of stepwise increments in gene numbers. Random forest is a widely used machine learning method that works by generating multiple bootstrapped versions of the training data, and fitting a decision tree to each of these bootstraps. In doing so, we settled on the number of genes that yielded a cross-validation misclassification error rate of less than 5%. Additionally, Brier scores and average probabilities were used as metrics to assist in selecting classifier size (Extended Data Fig. 1). We then used the resultant CTS classifier to perform random forest prediction of CTSs in the entire cohort using the random forest package in R. This approach was also done for samples from the RESERVE-U study (BioProject no. PRJNA794277)^25^ and VANISH trial (E-MTAB-7581)^13^. Posterior probabilities were calculated using the random forest package.

### Consensus clustering

Normalized gene expression data were used to conduct de novo consensus clustering using the ConsensusClusterPlus method^62^, as described previously^9^. Briefly, 7,260 unique gene expression profiles across 1,122 patient samples obtained on ICU admission were used. We selected the agglomerative hierarchical clustering algorithm on 1 minus Pearson correlation distances, 90% item (sample) resampling, 90% gene resampling, 1,000 iterations and a cluster range of *k* = 2–10. To estimate *k* (the number of subtypes), we combined empirical cumulative distribution functions, the area-under-cumulative distribution function curve, silhouette widths available in the cluster package and cophenetic distance correlation analysis to assess clustering stability. Silhouette widths^23^, presented for all cohorts, directly quantify subtype consistency, stability and classifier performance. The highest cophenetic correlation coefficient indicates the optimal cluster size.

### Pathway analysis, scRNA-seq integration and data mining

Gene expression data with CTS assignments was analyzed using the GSEA software available from the Broad Institute (www.gsea-msigdb.org/gsea/index.jsp)^63^. GSEA is a computational method that determines whether a set of genes defined a priori shows statistically significant and concordant differences between biological states (for example, phenotypes). We used the hallmarks molecular signatures for all analyses. An FDR-adjusted *P* < 0.05 defined significant overrepresentation. The computational method AUCell^28^ was used to evaluate the activity of gene sets at the single-cell level in the sepsis single-cell atlas^27^, facilitating the identification of cell-type-specific gene expression patterns. AUCell operates by computing the AUC for each gene set within each cell, based on the ranking of gene expression levels. This approach assesses the relative enrichment of a gene set, facilitating the identification of active biological processes in specific cellular subpopulations.

### Plasma biomarker assays

For the MARS cohort, measurements were done in EDTA anticoagulated plasma obtained within 16 h after ICU admission. IL-6, IL-8, IL-10 and soluble E-selectin were measured using the FlexSet Cytometric Bead Array (BD Biosciences) using FACSCalibur (BD Biosciences). NGAL, protein C, MMP8, tPA, PAI1, angiopoietin-1, angiopoietin-2 (all R&D systems) and D-dimer (Procartaplex, eBioscence) were measured using the Luminex multiplex assay with BioPlex 200 (Bio-Rad Laboratories). Protein biomarkers were selected because they reflect changes in pathophysiological domains considered important for the pathogenesis of sepsis^7,64,65^: activation of the cytokine network (IL-6, IL-8, IL-10); systemic inflammation (MMP8, NGAL); activation of coagulation and fibrinolysis (D-dimer, protein C, tPA, plasminogen activator inhibitor-1); and activation and dysfunction of the vascular endothelium (soluble E-selectin, angiopoietin-1 and angiopoietin-2). Additionally, while other biomarkers could have been selected, the chosen biomarkers could be measured in multiplex assays, allowing analyses in large patient groups.

### Statistics and reproducibility

We performed nonparametric tests for comparisons pertaining to continuous variables using a Kruskal–Wallis test, followed by a Dunn’s post hoc test; categorical data were compared using a chi-squared test and post hoc tests. Kaplan–Meier plots and log-rank tests were done using the survival R package. We assessed the impact of corticosteroid administration on 28-day mortality in MARS patients assigned to CTSs using propensity score matching using the MatchIt v.4.5.5 package in R. The treatment variable, corticosteroid use, was matched according to the SOFA score, hospital-to-ICU admission time interval, primary site of infection, septic shock and age. Propensity scores were estimated using a logistic regression model. Nearest-neighbor matching using a 1:1 ratio was implemented to establish balance between treated and untreated patients. A caliper (set at 1.2) was used to match patients with similar propensity scores, thereby minimizing imbalance. The final matched dataset was used to evaluate the interaction between corticosteroid use and CTS assignment on 28-day mortality through logistic regression. The VANISH trial data (E-MTAB-7581)^13^ were used in a logistic regression model evaluating the interaction of hydrocortisone treatment and CTS membership on 28-day mortality. Throughout, *P* < 0.05 demarcated significance unless stated otherwise. The ROC AUC analysis was performed using the pROC method in R. NRI was calculated using the nricens v.1.6 method in R. All analyses were carried out using RStudio (v.4.3.2). No statistical method was used to predetermine sample size. All data from the MARS, VANISH and RESERVE-U cohorts were included in the analysis. On the other hand, samples from the GAinS cohort that failed quality control in their original studies were excluded. The experiments were not randomized. Investigators were blinded to sample identifiers while classifying samples into their respective research groups: MARS1–4, SRS1 and 2, or the adaptive, coagulopathic and inflammopathic subtypes.

### Reporting summary

Further information on research design is available in the Nature Portfolio Reporting Summary linked to this article.

## Online content

Any methods, additional references, Nature Portfolio reporting summaries, source data, extended data, supplementary information, acknowledgements, peer review information; details of author contributions and competing interests; and statements of data and code availability are available at 10.1038/s41591-025-03964-5.

## Supplementary information

Supplementary Information Consortium and affiliations.

Reporting Summary

## Data Availability

All datasets used in the study are located in the public domain. The MARS data are available at the Gene Expression Omnibus under accession nos. GSE65682 and GSE134347. The GAinS datasets are available at the ArrayExpress of the European Bioinformatics Institute (accession nos. E-MTAB-4421 and E-MTAB-4451) and the European Genome-Phenome Archive (accession no. EGAD00001008730). The VANISH dataset is available at ArrayExpress (accession no. E-MTAB-7581). The RESERVE-U dataset is available at the Sequence Read Archive (BioProject no. PRJNA794277).

## Code availability

The R package developed to assign patients into CTSs is available via GitHub at https://github.com/bpscicluna/ConsensusTranscriptomicSubtype. The code used to identify the CTSs is available via GitHub at https://github.com/bpscicluna/Consensus-Transcriptomic-Subtypes-of-Sepsis/.
