# Supplementary material for: A consensus blood transcriptomic framework for sepsis
Source: Nat Med. 2025 Sep 30;31(12):4119–30. doi: 10.1038/s41591-025-03964-5 (PMC12705454; doi:10.1038/s41591-025-03964-5)
Supplement: Supplementary file 1 — Consortium and affiliations. [file 41591_2025_3964_MOESM1_ESM.pdf]

nature medicine

Supplementary information https://doi.org/10.1038/s41591-025-03964-5

A consensus blood transcriptomic framework for sepsis

In the format provided by the authors and unedited

Molecular diagnosis and Risk Stratification in Sepsis (MARS) consortium:

Amsterdam UMC location University of Amsterdam: Friso M. de Beer, Lieuwe D. J. Bos, Gerie J. Glas, Arie J. Hoogendijk, Roosmarijn T. M. van Hooijdonk, Janneke Horn, Mischa A. Huson, Laura R. A. Schouten, Marcus J. Schultz, Marleen Straat, Lonneke A. van Vught, Luuk Wieske, Maryse A. Wiewel, Esther Witteveen.

University Medical Center Utrecht: Marc J.M. Bonten, David S.Y. Ong, Jos F. Frencken, Peter M.C. Klein Klouwenberg, Maria E. Koster-Brouwer, Kirsten van de Groep, Diana M. Verboom

Genomic Adavnces in Sepsis (GAinS) consortium:

Nigel Webster[12], Helen Galley[12], Jane Taylor[12], Sally Hall[12], Jenni Addison[12], Sian Roughton[12], Heather Tennant[12], Achyut Guleri[13], Natalia Waddington[13], Dilshan Arawwawala[14], John Durcan[14], Alasdair Short[14], Karen Swan[14], Sarah Williams[14], Susan Smolen[14], Christine Mitchell-Inwang[14], Emily Errington[15], Maie Templeton[15], Pyda Venatesh[16], Geraldine Ward[16], Marie McCauley[16], Simon Baudouin[17,33], Charley Higham[17], Jasmeet Soar[19], Sally Grier[19], Elaine Hall1[9], Stephen Brett[19], David Kitson[19], Robert Wilson[19], Laura Mountford[19], Juan Moreno[19], Peter Hall[20], Jackie Hewlett[20], Christopher Garrard[21], Julian Millo[21], Duncan Young[21], Penny Parsons[21], Alex Smiths[21], Roser Faras-Arraya[21], Jasmeet Soar[22], Parizade Raymode[22], Jonathan Thompson[23], Sarah Bowrey[23], Sandra Kazembe[23], Natalie Rich[23], Prem Andreou[23], Dawn Hales[23], Emma Roberts[23], Simon Fletcher[24], Melissa Rosbergen[24], Georgina Glister[24], Jeronimo Moreno Cuesta[25], Julian Bion[26], Joanne Millar[26], Elsa Jane Perry[26], Heather Willis[26], Natalie Mitchell[26], Sebastian Ruel[26], Ronald Carrera[26], Jude Wilde[26], Annette Nilson[26], Sarah Lees[26], Atul Kapila[27], Nicola Jacques[27], Jane Atkinson[27], Abby Brown[27], Heather Prowse[27], Anton Krige[28], Martin Bland[28], Lynne Bullock[28], Donna Harrison[28], Gary Mills[29,30], John Humphreys[29,30], Kelsey Armitage[29, 30], Shond Laha[31], Jacqueline Baldwin[31], Angela Walsh[31], Nicola Doherty[31], Stephen Drage[32], Laura Ortiz-Ruiz de Gordoa[32], Sarah Lowes[32], Charley Higham[33], Helen Walsh[33], Verity Calder[33], Catherine Swan[33], Heather Payne[33], David Higgins[34], Sarah Andrews[34], Sarah Mappleback[34], Chris Garrard[35,36], D. Watson[35,36], Eleanor McLees[35,36], Alice Purdy[35,36], Martin Stotz[37], Adaeze Ochelli-Okpue[37], Stephen Bonner[38], Iain Whitehead[38], Keith Hugil[38], Victoria Goodridge[38], Louisa Cawthor[38], Martin Kuper[39], Sheik Pahary[39], Geoffrey Bellingan[40], Richard Marshall[40], Hugh Montgomery[40], Jung Hyun Ryu[40], Georgia Bercades[40], Susan Boluda[40], Andrew Bentley[41], Katie Mccalman[41], Fiona Jefferies[41], Andrew Kwok[1], Narelle Maugeri[1], Jayachandran Radhakrishnan[1], and Alice Allcock[1].

Affiliations 1 to 11 can be found on the first page of the paper.

[12]Aberdeen Royal Infirmary, Aberdeen AB25 2ZN, UK.

[13]Blackpool Victoria Hospital, Blackpool FY3 8NR, UK.

[14]Broomfield Hospital, Chelmsford CM1 7ET, UK.

[15]Charing Cross Hospital, London W6 8RF, UK.

[16]Coventry and Warwickshire University Hospital, Coventry CV2 2DX, UK.

[17]Freeman Hospital, Newcastle upon Tyne NE7 7DN, UK.

[18]Frenchay Hospital, Bristol, UK and Southmead Hospital, Bristol BS16 1JE, UK.

[19]Hammersmith Hospital, London W12 0HS, UK.

[20]Huddersfield Royal Infirmary, Huddersfield HD3 3EA, UK.

[21]John Radcliffe Hospital, Headington, Oxford OX3 9DU, UK.

[22]Kettering General Hospital, Kettering NN16 8UZ, UK.

[23]Leicester Royal Infirmary, Leicester LE1 5WW, UK.

[24]Norfolk and Norwich University Hospital, Norwich NR4 7UY, UK.

[25]North Middlesex Hospital, London N19 1QX, UK.

[26]Queen Elizabeth Hospital, Birmingham B15 2GW, UK.

[27]Royal Berkshire Hospital, Reading RG1 5AN, UK.

[28]Royal Blackburn Hospital, Blackburn BB2 3HH, UK.

[29]Royal Hallamshire Hospital, Sheffield S10 2JF, UK.

[30]Northern General Hospital, Sheffield S5 7AU, UK.

[31]Royal Preston Hospital, Preston PR2 9HT, UK.

[32]Royal Sussex County Hospital, Brighton BN2 5BE, UK.

[33]Royal Victoria Infirmary, Newcastle upon Tyne NE1 4LP, UK.

[34]Southend Hospital, Westcliff-on-Sea SS0 0RY, UK.

[35]St Bartholomew's Hospital, London EC1A 7BE, UK.

[36]Royal London Hospital, London E1 1FR, UK.

[37]St Mary's Hospital, London W2 1NY, UK.

[38]James Cook University Hospital, Middlesbrough TS4 3BW, UK.

[39]Whittington Hospital, London N18 5NF, UK.

[40]University College London Hospital, UCLH, London NW1 2BU, UK.

[41]Wythenshawe Hospital, Manchester M23 9LT, UK.
