# Supplementary material for: A consensus blood transcriptomic framework for sepsis
Source: Nat Med. 2025 Sep 30;31(12):4119–30. doi: 10.1038/s41591-025-03964-5 (PMC12705454; doi:10.1038/s41591-025-03964-5)
Supplement: Supplementary file 2 — Reporting Summary [file 41591_2025_3964_MOESM2_ESM.pdf]

Corresponding author(s): Brendon P. Scicluna PhD

Last updated by author(s): Aug 1, 2025

Reporting Summary

Nature Portfolio wishes to improve the reproducibility of the work that we publish. This form provides structure for consistency and transparency in reporting. For further information on Nature Portfolio policies, see our Editorial Policies and the Editorial Policy Checklist.

Statistics

For all statistical analyses, confirm that the following items are present in the figure legend, table legend, main text, or Methods section.

n/a	Confirmed	
☐	☒	The exact sample size (*n*) for each experimental group/condition, given as a discrete number and unit of measurement
☐	☒	A statement on whether measurements were taken from distinct samples or whether the same sample was measured repeatedly
☐	☒	The statistical test(s) used AND whether they are one- or two-sided<br>*Only common tests should be described solely by name; describe more complex techniques in the Methods section.*
☐	☒	A description of all covariates tested
☐	☒	A description of any assumptions or corrections, such as tests of normality and adjustment for multiple comparisons
☐	☒	A full description of the statistical parameters including central tendency (e.g. means) or other basic estimates (e.g. regression coefficient) AND variation (e.g. standard deviation) or associated estimates of uncertainty (e.g. confidence intervals)
☐	☒	For null hypothesis testing, the test statistic (e.g. *F*, *t*, *r*) with confidence intervals, effect sizes, degrees of freedom and *P* value noted<br>*Give P values as exact values whenever suitable.*
☒	☐	For Bayesian analysis, information on the choice of priors and Markov chain Monte Carlo settings
☐	☒	For hierarchical and complex designs, identification of the appropriate level for tests and full reporting of outcomes
☒	☐	Estimates of effect sizes (e.g. Cohen's *d*, Pearson's *r*), indicating how they were calculated

Our web collection on statistics for biologists contains articles on many of the points above.

Software and code

Policy information about availability of computer code

Data collection	All gene expression data used in the study were obtained from the public domain. No software was used for data collection.
Data analysis	R packages:<br>Surrogate Variable Analysis (SVA) version 3.52.0 (https://code.bioconductor.org/browse/sva/)<br>BiocParallel version 1.38.0 (https://bioconductor.org/packages/BiocParallel/)<br>genefilter version 1.86.0 (https://bioconductor.org/packages/genefilter/)<br>Biostrings version 2.72.1 (https://bioconductor.org/packages/Biostrings/)<br>survival version 3.5-8 (CRAN repository)<br>dplyr version 1.1.4 (CRAN repository)<br>jaccard version 0.1.0 (CRAN repository)<br>dunn.test version 1.3.6 (CRAN repository)<br>MCL version 1.0 (CRAN repository)<br>cluster version 2.1.6 (CRAN repository)<br>CMA version 1.62.0 (https://bioconductor.org/packages/CMA/)<br>ConsensusClusterPlus version 1.68.0 (https://bioconductor.org/packages/ConsensusClusterPlus/)<br>AUCell version 1.30.1 (https://bioconductor.org/packages/AUCell/)<br>MatchIt version 4.5.5 (CRAN repository)<br>nricens version 1.6 (CRAN repository)<br>randomForest version 4.7-1.1 (CRAN repository)<br>ggplot2 version 3.5.1 (CRAN repository)<br>mixOmics version 6.28.0 (https://bioconductor.org/packages/release/bioc/html/mixOmics.html)

Cytoscape version 3.9.1 (https://cytoscape.org/)
GSEA version 4.3.2 (https://www.gsea-msigdb.org/gsea/index.jsp)

The R package developed to assign patients into Consensus Transcriptomic Subtypes is available for download at https://github.com/bpscicluna/ConsensusTranscriptomicSubtype. The code used to identify consensus transcriptomic subtypes is located at https://github.com/bpscicluna/Consensus-Transcriptomic-Subtypes-of-Sepsis/

For manuscripts utilizing custom algorithms or software that are central to the research but not yet described in published literature, software must be made available to editors and reviewers. We strongly encourage code deposition in a community repository (e.g. GitHub). See the Nature Portfolio guidelines for submitting code & software for further information.

Data

Policy information about availability of data

All manuscripts must include a data availability statement. This statement should provide the following information, where applicable:

- Accession codes, unique identifiers, or web links for publicly available datasets
- A description of any restrictions on data availability
- For clinical datasets or third party data, please ensure that the statement adheres to our policy

All gene expression data used in the study were obtained from the public domain. Specifically, GSE65682, GSE134347, E-MTAB-4421/E-MTAB-4451, EGAD00001008730, PRJNA794277 and E-MTAB-7581. The MARS and GAinS consortiums are committed to sharing, upon reasonable request from qualified scientific and medical researchers, patient-level clinical data, study-level clinical data and protocols after approval by a scientific review panel.

Human research participants

Policy information about studies involving human research participants and Sex and Gender in Research.

Reporting on sex and gender	Throughout the study, sex was reported. Analyses were adjusted for sex where applicable.
Population characteristics	Public blood gene expression datasets were used in the study. The MARS and GAinS studies included critically ill patients with all-cause sepsis and pneumonia or fecal peritonitis, respectively. The VANISH clinical trial included patients with septic shock. Only adult patients were included in the studies.
Recruitment	The MARS and GAinS studies prospectively included patients as part of observational study designs. The VANISH trial was a factorial (2x2), multicenter, double-blind, randomized clinical trial.
Ethics oversight	All samples were collected in accordance with respective local Intitutional Review Boards. The MARS patients were included via an opt-out consent method approved by the institutional review boards of both recruiting hospitals, that is, Amsterdam UMC and UMC Utrecht (IRB No. 10-056C). For GAinS, ethics approval was granted nationally and locally, with informed consent obtained from all patients or their legal representative and conducted under Research Ethics Committee approvals 05/MRE00/38, 08/H0505/78, and 06/Q1605/55. The VANISH trial was approved by the Oxford A research ethics committee, and written consent was obtained from patients or their legal representatives.

Note that full information on the approval of the study protocol must also be provided in the manuscript.

Field-specific reporting

Please select the one below that is the best fit for your research. If you are not sure, read the appropriate sections before making your selection.

☒ Life sciences ☐ Behavioural & social sciences ☐ Ecological, evolutionary & environmental sciences

For a reference copy of the document with all sections, see nature.com/documents/nr-reporting-summary-flat.pdf

Life sciences study design

All studies must disclose on these points even when the disclosure is negative.

Sample size	Sample sizes were not determined a priori. Publicly available data of 1122 patients on admission (MARS and GAinS), 817 patient samples of the GAinS cohort obtained on days 3 and 5 after ICU admission, 176 VANISH trial patients and 128 RESERVE-U patient samples were included.
Data exclusions	Patient samples obtained from the GAinS cohort that did not pass RNA-seq quality control were excluded.
Replication	Consensus transcriptomic subtypes were derived using cross-validation statistics (MARS and GAinS), and validated in an independent cohort (VANISH and RESERVE-U studies)
Randomization	Data was initially handled according to study (MARS and GAinS). We assessed the impact of corticosteroid administration on 28-day mortality among MARS patients assigned to consensus transcriptomic subtypes utilizing propensity score matching. The treatment variable, corticosteroid use, was matched according to the SOFA score, primary site of infection, septic shock, and age. Validation of the consensus

transcriptomic subtypes was conducted in two independent cohorts of patients included in (1) the VANISH trial, which was a multicenter, double-blind, randomized clinical trial, and (2) the Research in the Epidemiology of Severe and Emerging Infections in Uganda (RESERVE-U) study.

Blinding

Consensus transcriptomic subtypes were derived blinded to clinical characteristics, treatment, and outcomes.

Reporting for specific materials, systems and methods

We require information from authors about some types of materials, experimental systems and methods used in many studies. Here, indicate whether each material, system or method listed is relevant to your study. If you are not sure if a list item applies to your research, read the appropriate section before selecting a response.

Materials & experimental systems

n/a	Involved in the study	
☒	☐	Antibodies
☒	☐	Eukaryotic cell lines
☒	☐	Palaeontology and archaeology
☒	☐	Animals and other organisms
☐	☒	Clinical data
☒	☐	Dual use research of concern

Methods

n/a	Involved in the study	
☒	☐	ChIP-seq
☒	☐	Flow cytometry
☒	☐	MRI-based neuroimaging

Clinical data

Policy information about clinical studies

All manuscripts should comply with the ICMJE guidelines for publication of clinical research and a completed CONSORT checklist must be included with all submissions.

Clinical trial registration

MARS (NCT01905033), GAinS (NCT00121196), and VANISH (ISRCTN 20769191).

Study protocol

N/A - All study cohorts were previously published

Data collection

The MARS study, a prospective observational study in the mixed ICUs of two tertiary teaching hospitals in the Netherlands (Academic Medical Center, Amsterdam and University Medical Center Utrecht, Utrecht) enrolled consecutive patients with sepsis who were older than 18 years of age and had been admitted to the ICU between January 2011 and January 2014. The GAinS study, recruited patients (>18 years old) from 34 intensive care units (ICUs) across the United Kingdom between 16/11/2005 and 30/05/2018.

Outcomes

The primary outcome was identification of a consensus blood transcriptomic signature between three distinct subclassification methods. This was achieved by unsupervised clustering, network analysis and hypergeometric tests. Subsequent to the identification of a common signature, consensus transcriptomic subtypes were analyzed against clinical severity scores, primary sites of infection, corticosteroid treatment, and mortality.
